# Exacerbated Activation of the NLRP3 Inflammasome in the Placentas from Women Who Developed Chronic Venous Disease during Pregnancy

**DOI:** 10.3390/ijms25105528

**Published:** 2024-05-18

**Authors:** María Asunción Sánchez-Gil, Oscar Fraile-Martinez, Cielo García-Montero, Diego De Leon-Oliva, Diego Liviu Boaru, Patricia De Castro-Martinez, Adrían Camacho-Alcázar, Juan A. De León-Luis, Coral Bravo, Raúl Díaz-Pedrero, Laura López-Gonzalez, Julia Bujan, María J. Cancelo, Melchor Álvarez-Mon, Natalio García-Honduvilla, Miguel A. Saez, Miguel A. Ortega

**Affiliations:** 1 Department of Medicine and Medical Specialities, Faculty of Medicine and Health Sciences, University of Alcalá, 28801 Alcala de Henares, Spain; msangil@oc.mde.es (M.A.S.-G.); oscar.fraile@uah.es (O.F.-M.); cielo.garciamontero@uah.es (C.G.-M.); diegodleonoliva01@gmail.com (D.D.L.-O.); diego.boaru@edu.uah.es (D.L.B.); patriciadecastro1999@gmail.com (P.D.C.-M.); adrianmanuelcamacho@gmail.com (A.C.-A.); mjulia.bujan@uah.es (J.B.); mademons@gmail.com (M.Á.-M.); natalio.garcia@uah.es (N.G.-H.); msaega1@oc.mde.es (M.A.S.); 2 Ramón y Cajal Institute of Sanitary Research (IRYCIS), 28034 Madrid, Spain; raul.diazp@uah.es (R.D.-P.); laura.lgonzalez@uah.es (L.L.-G.); 3 University Defense Center of Madrid (CUD), 28047 Madrid, Spain; 4 Department of Obstetrics and Gynecology, University Hospital Gregorio Marañón, 28009 Madrid, Spain; jaleon@ucm.es (J.A.D.L.-L.); cbravoarribas@gmail.com (C.B.); 5 Health Research Institute Gregorio Marañón, 28009 Madrid, Spain; 6 Department of Surgery, Medical and Social Sciences, Faculty of Medicine and Health Sciences, University of Alcalá, 28801 Alcala de Henares, Spain; mjesus.cancelo@uah.es; 7 Pathological Anatomy Service, University Hospital Gómez-Ulla, 28806 Alcala de Henares, Spain; 8 Department of Obstetrics and Gynecology, University Hospital of Guadalajara, 19002 Guadalajara, Spain; 9 Immune System Diseases-Rheumatology and Internal Medicine Service, University Hospital Prince of Asturias, Networking Research Center on for Liver and Digestive Diseases (CIBEREHD), 28806 Alcala de Henares, Spain

**Keywords:** placenta, chronic venous disease (CVD), NLRP3 inflammasome, ASC, caspases, interleukin IL-1β

## Abstract

Chronic venous disease (CVD) comprises a spectrum of morphofunctional disorders affecting the venous system, affecting approximately 1 in 3 women during gestation. Emerging evidence highlights diverse maternofetal implications stemming from CVD, particularly impacting the placenta. While systemic inflammation has been associated with pregnancy-related CVD, preliminary findings suggest a potential link between this condition and exacerbated inflammation in the placental tissue. Inflammasomes are major orchestrators of immune responses and inflammation in different organs and systems. Notwithstanding the relevance of inflammasomes, specifically the NLRP3 (nucleotide-binding domain, leucine-rich-containing family, pyrin domain-containing-3)- which has been demonstrated in the placentas of women with different obstetric complications, the precise involvement of this component in the placentas of women with CVD remains to be explored. This study employs immunohistochemistry and real-time PCR (RT-qPCR) to examine the gene and protein expression of key components in both canonical and non-canonical pathways of the NLRP3 inflammasome (NLRP3, ASC—apoptosis-associated speck-like protein containing a C-terminal caspase recruitment domain—caspase 1, caspase 5, caspase 8, and interleukin 1β) within the placental tissue of women affected by CVD. Our findings reveal a substantial upregulation of these components in CVD-affected placentas, indicating a potential pathophysiological role of the NLRP3 inflammasome in the development of this condition. Subsequent investigations should focus on assessing translational interventions addressing this dysregulation in affected patient populations.

## 1. Introduction

Chronic venous disease (CVD) encompasses functional and structural alterations in the venous system triggered by ambulatory venous hypertension and mainly affects the lower extremities, with the most characteristic clinical manifestations being edema, heaviness, varicose veins, and telangiectasia [1]. The etiology of increased venous pressure in CVD appears to involve a complex interplay of genetic and environmental factors [2]. It is estimated that one in three women will develop some sign of CVD during pregnancy, and up to 15% of them will present evidence of varicose veins (VVs), with the risk escalating with each subsequent pregnancy [3,4]. The emergence of CVD in pregnancy is attributed to elevated venous pressure in the lower limbs, stemming from compression of the inferior vena cava and iliac veins by the gravid uterus, along with heightened venous distensibility due to hormonal fluctuations [5]. Previous findings indicate that suffering from CVD during this period can result in a significant stressor for maternofetal structures, with the placenta and the umbilical cord showing evidence of augmented pathological processes like oxidative stress, hypoxia, and abnormal angiogenesis and lymphangiogenesis [6,7]. However, the extent and possible pathobiological involvement of these changes in maternofetal structures need to be characterized more deeply.

Strong evidence underscores the association between CVD and inflammation, highlighting its potential impact on both maternal and fetal health. For instance, previous research has established a correlation between CVD and heightened systemic levels of circulating proinflammatory markers, impacting both the maternal and fetal compartments [8]. This systemic proinflammatory milieu exerts discernible effects on critical maternofetal structures such as the placenta and the umbilical cord [9,10]. In this sense, emerging evidence suggests a potential interplay between hyperactivated inflammasomes and altered immunoinflammatory responses within affected placental tissues under various obstetric complications [11]. However, the precise involvement and significance of inflammasomes, particularly the NLRP3 inflammasome nucleotide-binding domain, leucine-rich-containing family, pyrin domain-containing-3-, in the context of CVD-affected placentas remain incompletely understood. Notably, inflammasomes, including NLRP3, are intracellular multiprotein complexes crucial for mediating inflammatory responses by stimulating caspase 1 and then leading to the production of proinflammatory cytokines such as interleukin 1β and interleukin 18 [12]. Indeed, we had previously demonstrated that IL-18 is upregulated in the placental tissue of women with CVD [13]. Dysregulation of the NLRP3 inflammasome pathway has been implicated in various pathological conditions, including inflammatory disorders, autoimmune diseases, and pregnancy-related complications, underlining its potential significance in elucidating the inflammatory status and pathology associated with CVD during pregnancy [11,14].

This study aimed to investigate the gene and protein expression levels of pivotal components involved in both canonical and non-canonical pathways of the NLRP3 inflammasome, including NLRP3, ASC, and apoptosis-associated speck-like protein containing a C-terminal caspase recruitment domain, caspase 1, caspase 5, caspase 8, and interleukin 1β, within the placental tissue of women afflicted by CVD. Immunohistochemistry (IHC) and real-time PCR (RT-qPCR) were employed to comprehensively assess the molecular alterations associated with CVD in the placenta, shedding light on the potential role of the NLRP3 inflammasome pathway in the pathophysiology of this condition during pregnancy. In parallel, we will compare the gene and protein expression of these components in healthy pregnant women (HC) with no diagnosed pathologies.

## 2. Results

### 2.1. The Placentas of Women with CVD Exhibit Increased Expression of Canonical Inflammasome Components

**Figure 1:**
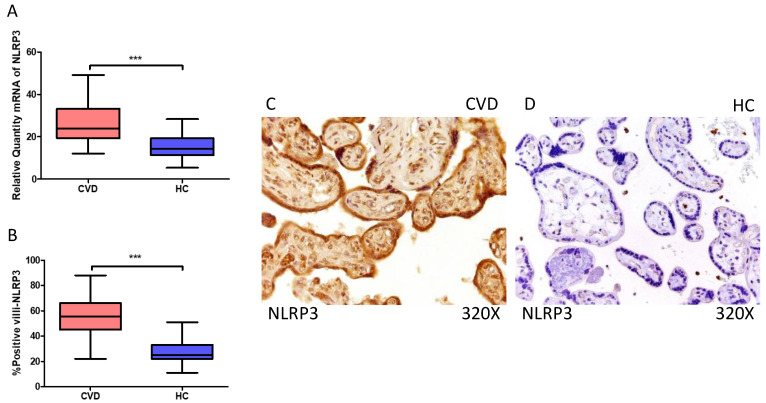
(**A**) NLRP3 mRNA expression in women with CVD and HC (*** *p* < 0.0001). (**B**) IRS scores for NLRP3 expression in the placental villi of the CVD and HC groups (**C**,**D**) (*** *p* < 0.0001). Images showing immunostaining for NLRP3 in the placental villi of the CVD and HC.

Firstly, our findings demonstrate a statistically significant increase in NLRP3 gene expression (RT-qPCR) in placental tissue of pregnant women with CVD (*** *p* < 0.0001; CVD = 23.906 [12.061–49.132], HC = 14.336 [5.351–28.317], Figure 1A). Histological analysis of placental villi revealed a significant upregulation of NLRP3 protein expression (%) in chorionic villi from women with CVD (*** *p* < 0.0001; CVD = 55.500 [22.000–91.000], HC = 27.000 [14.000–44.000], Figure 1B). Tissue expression of NLRP3 was prominently observed in all placental villi of women affected by CVD compared to HC, particularly within the syncytiotrophoblast layer (Figure 1C,D).

**Figure 2:**
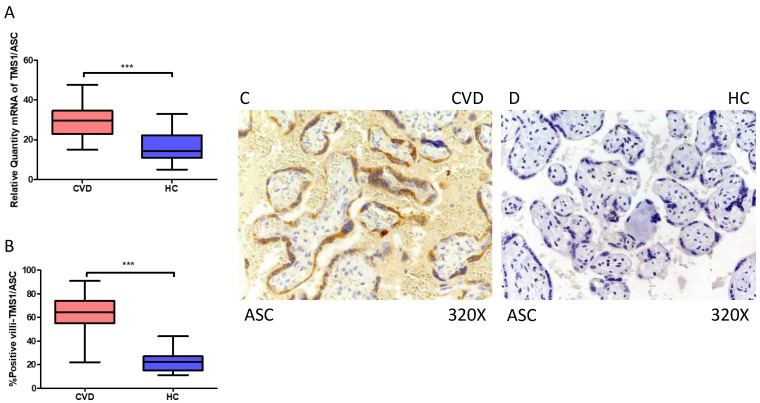
(**A**) ASC mRNA expression in women with CVD and HC (*** *p* < 0.0001). (**B**) IRS scores for ASC expression in the placental villi of the CVD and HC groups (**C**,**D**) (*** *p* < 0.0001). Images showing immunostaining for ASC in the placental villi of the CVD and HC.

In the same line, our data indicate a statistically significant increase in ASC gene expression (RT-qPCR) in placental tissue of pregnant women with CVD (*** *p* < 0.0001; CVD = 29.634 [15.065–47.652], HC = 14.312 [5.022–32.945], Figure 2A). Histological examination of placental villi displayed a significant elevation in ASC protein expression (%) in chorionic villi from women with CVD (*** *p* < 0.0001; CVD = 64.500 [22.000–91.000], HC = 22.500 [11.000–44.000], Figure 2B). Tissue expression of ASC was markedly heightened in all placental villi of women affected by CVD in comparison to HC, particularly within the syncytiotrophoblast layer (Figure 2C,D).

**Figure 3:**
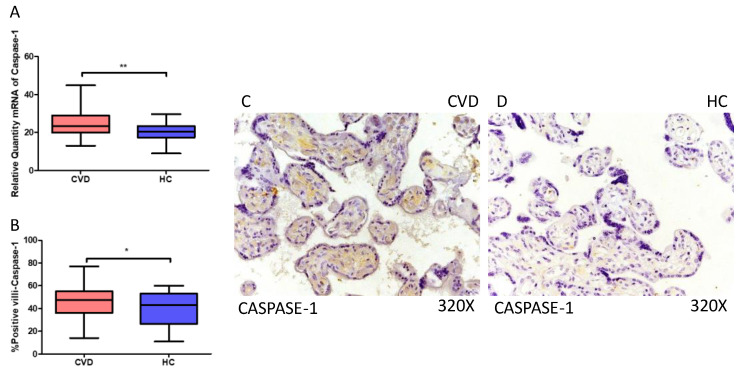
(**A**) Caspase-1 mRNA expression in women with CVD and HC (** *p* = 0.0014). (**B**) IRS scores for caspase-1 expression in the placental villi of the CVD and HC groups (**C**,**D**) (* *p* = 0.0187). Images showing immunostaining for caspase-1 in the placental villi of the CVD and HC.

Moreover, our results suggest a statistically significant increase in caspase 1 gene expression (RT-qPCR) in placental tissue of pregnant women with CVD (** *p* = 0.0014; CVD = 23.381 [12.981–44.856], HC = 20.539 [8.962–29.672], Figure 3A). Histological analysis of placental villi revealed a notable rise in caspase 1 protein expression (%) in chorionic villi from women with CVD (* *p* = 0.0187; CVD = 47.500 [14.000–77.000], HC = 43.000 [11.000–60.000], Figure 3B). Tissue expression of caspase 1 was significantly elevated in all placental villi of women affected by CVD compared to HC, particularly within the syncytiotrophoblast layer (Figure 3C,D).

**Figure 4:**
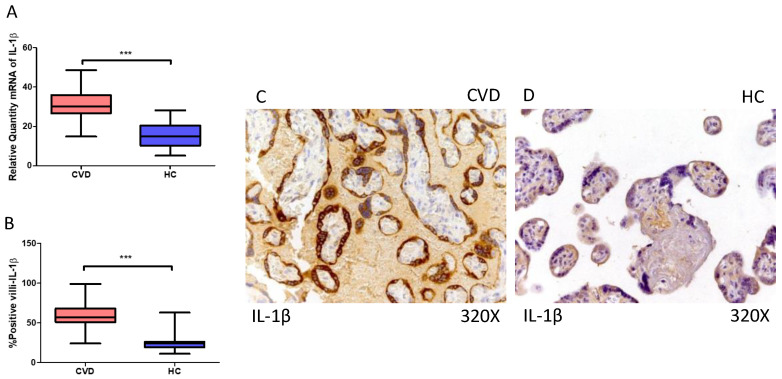
(**A**) IL-1β mRNA expression in women with CVD and HC (*** *p* < 0.0001). (**B**) IRS scores for IL-1β expression in the placental villi of the CVD and HC group (**C**,**D**) (*** *p* < 0.0001). Images showing immunostaining for IL-1β in the placental villi of the CVD and HC.

Furthermore, our findings indicate a statistically significant increase in IL-1β gene expression (RT-qPCR) in the placental tissue of pregnant women with CVD (*** *p* < 0.0001; CVD = 30.047 [14.846–48.617], HC = 14.988 [5.066–28.156], Figure 4A). Histological examination of placental villi showed a marked elevation in IL-1 beta protein expression (%) in chorionic villi from women with CVD (*** *p* < 0.0001; CVD = 57.000 [24.000–99.000], HC = 24.000 [11.000–63.000], Figure 4B). Tissue expression of IL-1β was significantly heightened in all placental villi of women affected by CVD compared to HC, particularly within the syncytiotrophoblast layer (Figure 4C,D).

### 2.2. The Placentas of Women with CVD Display Enhanced Expression of Non-Canonical Caspase-5 and Caspase-8

**Figure 5:**
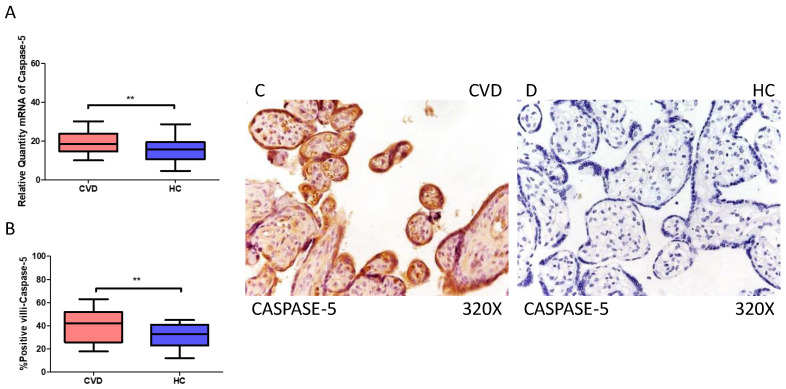
(**A**) Caspase-5 mRNA expression in women with CVD and HC (** *p* = 0.0013). (**B**) IRS scores for caspase-5 expression in the placental villi of the CVD and HC groups (**C**,**D**) (** *p* = 0.0010). Images showing immunostaining for caspase-5 in the placental villi of the CVD and HC.

On the other hand, our findings suggest a statistically significant increase in caspase 5 gene expression (RT-qPCR) in placental tissue of pregnant women with CVD (** *p* = 0.0013; CVD = 18.562 [9.968–30.165], HC = 15.611 [4.561–28.652], Figure 5A). Histological examination of placental villi showed a notable rise in caspase 5 protein expression (%) in chorionic villi from women with CVD (** *p* = 0.0010; CVD = 42.000 [18.000–63.100], HC = 33.000 [12.000–45.000], Figure 5B). Tissue expression of caspase 5 was significantly elevated in all placental villi of women affected by CVD compared to HC, particularly within the syncytiotrophoblast layer (Figure 5C,D).

**Figure 6:**
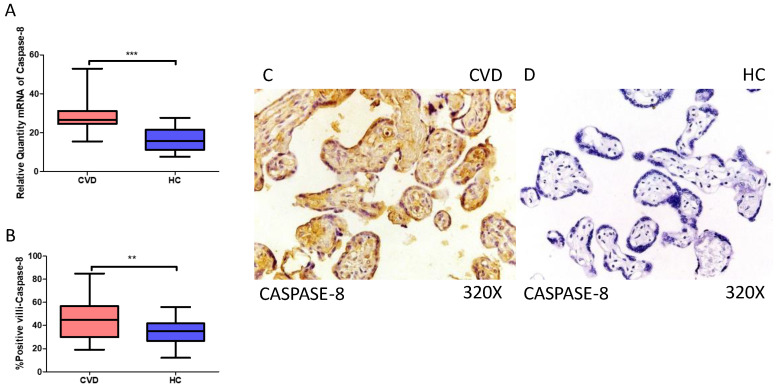
(**A**) Caspase-8 mRNA expression in women with CVD and HC (*** *p* < 0.0001). (**B**) IRS scores for caspase-8 expression in the placental villi of the CVD and HC groups (**C**,**D**) (** *p* = 0.0019). Images showing immunostaining for caspase-8 in the placental villi of the CVD and HC.

Simultaneously, our results indicate a statistically significant increase in caspase 8 gene expression (RT-qPCR) in placental tissue of pregnant women with CVD (*** *p* < 0.0001; CVD = 26.565 [15.566–52.948], HC = 15.829 [7.652–27.652], Figure 6A). Histological analysis of placental villi displayed a notable elevation in caspase 8 protein expression (%) in chorionic villi from women with CVD (** *p* = 0.0019; CVD = 45.000 [19.000–85.000], HC = 35.000 [12.000–56.000], Figure 6B). Tissue expression of caspase 8 was significantly elevated in all placental villi of women affected by CVD (Figure 6C,D).

## 3. Discussion

The findings of the present study shed light on the intricate relationship between CVD and inflammation, particularly within the context of pregnancy [15,16]. CVD, characterized by functional and structural alterations in the venous system due to ambulatory venous hypertension, is hypothesized to be primarily an inflammatory condition [17]. This hypothesis is particularly relevant during pregnancy, as the demanding physiological changes impose significant stress on the vascular system, potentially exacerbating inflammatory processes that may affect both the mother and the fetus [8,18]. Our study delved into the molecular mechanisms underlying CVD-associated inflammation by examining the gene and protein expression of key components of the NLRP3 inflammasome within placental tissue. The elevated expression of NLRP3, ASC, and caspases 1, 5, and 8, alongside increased levels of IL-1β, underscores the activation of both canonical and non-canonical inflammasome pathways in response to CVD-related stressors. These findings support the existing literature implicating inflammation in the pathogenesis of CVD and highlight the placenta as a site of active inflammatory processes in pregnancies affected by vascular disorders [13,19,20].

The placenta is an organ that constitutively expresses NLRP3 and other inflammasomes [14]. In vitro studies have defined that trophoblasts and other placental cells expressed NLRP1, NLRP3, NLRC4, and NLRP7 inflammasomes in the first trimester of pregnancy, and some of them also in association with parturition [11]. Circulating leukocytes of pregnant women also exhibit enhanced expression of the NLRP3 inflammasome as pregnancy progresses [21]. The NLRP3 inflammasome plays a pivotal role in mediating inflammatory responses, as it is known that it is a key regulator of the immune response and is associated with various high-risk reproductive disorders like endometriosis and polycystic ovarian syndrome, as well as those related to pregnancy like gestational diabetes, preeclampsia, preterm birth, and recurrent spontaneous abortion [22]. Mechanistically, the NLRP3 inflammasome is regulated by canonical and non-canonical routes. The former comprise a two-step process that begins with the recognition of damage-associated molecular patterns (DAMPs) and pathogen-associated molecular patterns (PAMPs), which then bind to their receptors to facilitate the translocation of nuclear factor kappa beta (NF-κB) into the nucleus, where it activates the transcription of NLRP3, caspase 1, IL-1β, and IL-18 [23]. Then, step 2 begins with NLRP3 detecting danger signals, which cause the NLRP3 inflammasome to become activated by potassium K+ efflux, causing NLRP3 and ASC oligomerization and assembly. The hyperactivation of the NLRP3 inflammasome in the placentas of women with CVD could be related to previous observations reporting that this tissue exhibits different markers of tissue stress and damage [18,24,25]. The NLRP3 inflammasome is mainly activated through the dysregulation of ionic balance, oxidative stress, mitochondrial dysfunction, and lysosomal damage [26]. Despite the fact that no studies have specifically focused on lysosomal damage and ionic imbalance, our past works have demonstrated that placental tissue and other maternofetal structures have evidence of increased oxidative stress, with significant consequences for both the mother and fetus [25,27]. Therefore, oxidative stress could be a potential link between CVD and NLRP3 activation in the placental tissue, although other established mechanisms observed in previous studies like hypoxia [24] or alterations in molecular routes like PI3K/Akt/mTOR [9] could also lead to enhanced activation of the NLRP3 inflammasome [28,29]. Future studies should be directed to uncover the possible NLRP3 activators in the placentas of women with CVD.

On the other hand, ASC is an adaptor protein that interacts with several inflammasomes, such as NLRP3, and that is expressed in several tissues, including the trophoblasts and other cells located in the placenta [21,30]. The interaction of NLRP3 with ASC is central to the formation of functional NLRP3 inflammasomes and the induction of caspase 1 activation [31]. Caspases are enzymes involved in various cellular processes, such as embryonic development, cell differentiation, and tissue homeostasis [32]. Their activity is regulated post-translationally, allowing for rapid responses to cellular stress and pathogens [33]. Although research is still limited about the involvement of caspases in pregnancy-related inflammatory disorders, it has already been explored that caspase 1 can play a role in the specific context of preterm birth and maternal metabolic disorders [34]. Also, this marker has been found to be involved in parturition, with higher concentrations in the amniotic fluid of women in labor [35]. Therefore, our study suggests that caspase 1 activation can be significantly implicated in the pathogenesis of CVD in association with the functional activation of the NLRP3 inflammasome, as measured by its interaction with ASC.

The activation of caspase 1 then leads to the cleavage of pro-IL-1β, pro-IL-18, and gasdermin D (GSDMD), responsible for forming a transmembrane pore that facilitates the secretion of active IL-1β and IL-18 [36,37]. IL-1β and IL-18 are two members of the IL-1 family that play several biological roles in various tissues, including the coordination of innate and adaptive immune responses and the regulation of cellular metabolism [38]. Abnormal levels of these cytokines have been linked to the presence of hypertension during pregnancy [39], whereas their overexpression in the placenta has been linked to a plethora of obstetric complications [40,41,42]. Also, the formation of active pores due to GSDMD and the release of IL-1β and IL-18 are responsible for a special type of inflammatory cell death named pyroptosis, whose relevance in physiological and pathological conditions is the subject of various studies [43,44]. Enhanced placental pyroptosis has been suggested as a central feature in the pathogenesis of early-onset preeclampsia, leading to the release of different factors into the maternal circulation that contribute to systemic inflammation [45]. This mechanism seems to be particularly important in the Hofbauer cells, which are responsible for mediating inflammatory processes in the placenta [46]. Therefore, our results suggest that pyroptosis could be an important pathological event occurring in the placental tissue of women with CVD, although its precise consequences should be explored in further work.

Importantly, not only caspase 1, but also different types of caspases like caspase 5 and caspase 8 are able to induce pyroptosis by acting through non-canonical NLRP3 inflammasome pathways [44,47]. Caspase 5 can directly cause the production of IL-1β and IL-18 when it is exposed to PAMPs such as lipopolysaccharides and DAMPs acting through two major pathways: (a) Cleaving GSDMD and opening membrane pores to cause pyroptotic cell death, or (b) activating the NLRP3 inflammasome [48,49,50]. Caspase 8 is activated in response to cell surface Death Receptors (DRs) like FAS, TRAIL-R, and TNF-R, triggering death receptor-mediated apoptosis and also other processes like anoikis, autophagy, and pyroptosis [47]. Previous works have found that caspase-8 can directly regulate IL-1β independent of inflammasomes or indirectly through the regulation of inflammasomes, depending on the stimuli that initiate the signaling cascade [51]. It has been documented in previous studies [52,53] that women with various obstetric problems, such as preterm labor and preeclampsia, have altered expression of caspase 8. Likewise, we also recently observed that not only caspase 8 but also caspase 5 were enhanced in the placental tissue of women with late-onset preeclampsia [54]. In agreement with these studies, we propose that caspase 5 and caspase 8 could be two important agents involved in the pathogenesis of CVD in the placental tissue of affected women.

Overall, our study suggests that NLRP3 hyperactivation and other related processes like pyroptosis could be an important inflammatory mechanism related to CVD during pregnancy, although further work is warranted to deepen the relationship between this condition and the NLRP3 inflammasome. Moreover, our study underscores the importance of exploring novel therapeutic targets aimed at modulating inflammasome activation to mitigate the inflammatory burden associated with CVD during pregnancy. Future research endeavors should focus on elucidating the mechanistic intricacies of inflammasome-mediated inflammation in CVD, paving the way for the development of targeted interventions aimed at alleviating maternal and fetal morbidity associated with this prevalent vascular disorder.

## 4. Materials and Methods

### 4.1. Study Design and Participants

An analytical and observational study was prospectively conducted involving 114 pregnant women in the third trimester. Among these, 52 women constituted the HC group, while 62 women were diagnosed with chronic venous disease (CVD) based on CEAP categorization [55]. The present study meticulously adhered to fundamental ethical principles, including autonomy, beneficence, non-maleficence, and distributive justice. Furthermore, it strictly followed the guidelines outlined in Good Clinical Practice as well as the ethical standards delineated in the most recent Helsinki Declaration (2013) and Oviedo Convention (1997). Prior to participation, all patients were comprehensively informed, and each provided written consent voluntarily. Approval for the study was obtained from the Clinical Research Ethics Committee of the Central University Hospital of Defense University of Alcalá (37/17). The medical records were thoroughly examined, and the physical well-being of participants was assessed during the third-trimester consultation. Additionally, ultrasound evaluations of the lower extremities were conducted at a frequency of 7.5 MHz using an Eco-Doppler device (Portable M-Turbo Eco-Doppler; SonoSite, Inc., Bothell, WA, USA).

Our study enrolled women aged over 18 exhibiting third-trimester clinical manifestations of venous disease in the lower extremities, meeting the inclusion criteria stipulated by the clinical–etiological–anatomical–pathophysiological (CEAP) classification ≥ 1. Exclusion criteria comprised individuals previously diagnosed with hypertension, venous malformations, cardiac, renal, or pulmonary insufficiency, autoimmune disorders, a body mass index (BMI) below 25, diabetes mellitus, gestational diabetes mellitus, or other endocrine disorders, active infectious diseases, engagement in toxicological habits (e.g., cannabis, heroin, cocaine, or amphetamines), preeclampsia and/or HELLP syndrome, alcohol consumption less than one unit per day, tobacco use less than one cigarette per day, identifiable causes of intrauterine growth restriction, pathological placental conditions such as infarction, avascular villi, delayed villous maturation, or chronic villitis, and any subsequent development of exclusionary criteria until delivery.

Utilizing the same sample cohort as previously reported [56] and summarized in Table 1, women in the HC group exhibited a median gestational age of 34 years (interquartile range (IQR), 27–41 years) and a median gestational duration of 41 weeks (IQR, 39–42 weeks). Conversely, women diagnosed with CVD had a median gestational age of 33 years (IQR, 22–40 years) and a median gestational duration of 40.5 weeks (IQR, 39–41.5 weeks). Notably, there were no significant disparities in the number of prior pregnancies between the groups: 19 (36.5%) for HC women and 33 (53.2%) for those with CVD (Table 1). Clinical parameters such as gestational age, mode of delivery (cesarean section), history of prior pregnancies, previous abortions, regularity of menstrual cycles, and occupational status (sedentary, as shown in Table 1) did not exhibit statistically significant differences between the CVD and HC groups.

### 4.2. Sample Collection and Processing

Placental biopsies were conducted on a cohort of 114 patients at the delivery moment. Each placenta was sectioned into five pieces, each comprising distinct cotyledon compositions, utilizing a surgical knife for separation. Subsequently, two sterile tubes were employed to contain placental fragments, one filled with RNAlater^®^ solution (Ambion; Thermo Fisher Scientific, Inc., Waltham, MA, USA) and the other with Minimum Essential Medium (MEM; Thermo Fisher Scientific, Inc., Waltham, MA, USA) supplemented with 1% antibiotic/antimycotic solution (Streptomycin, Amphotericin B, and Penicillin; Thermo Fisher Scientific, Inc.). Under aseptic conditions within a class II laminar flow hood (Telstar AV 30/70 Müller 220 V 50 MHz; Telstar; Azbil Corporation, Chiyoda-ku, Tokyo, Japan), the samples were processed and preserved for subsequent gene expression analysis. Specifically, the placental fragments were submerged in 1 mL of RNAlater^®^ solution and stored at −80 °C. Placental samples preserved in MEM were designated for histological and immunohistochemical investigations.

To vanish erythrocytes, MEM devoid of antibiotics was utilized to rehydrate the samples iteratively. Subsequently, the samples were sectioned into 2 cm lengths and fixed in F13 solution according to standardized protocols (comprising 60% ethanol, 20% methanol, 7% polyethylene glycol, and 13% distilled water) [56]. Following fixation, the samples were embedded in paraffin using molds. Upon solidification of the paraffin, sections of 5 μm thickness were obtained utilizing an HM 350 S rotation microtome (Thermo Fisher Scientific, Inc., Waltham, MA, USA). These sections were then immersed in a hot water bath and mounted onto glass slides previously treated with 10% polyLys to enhance adhesion.

### 4.3. Protein Expression by Immunohistochemistry Assays

Consistent with prior methodologies [56], the avidin–biotin complex technique employing avidin peroxidase was utilized to detect antigen–antibody reactions. Immunohistochemical analyses were performed on paraffin-embedded placental specimens (with details of specific antibodies provided in Table 2 of the protocol).

The primary antibody was applied to placental samples for a duration of 90 min, followed by an overnight incubation with 3% BSA Blocker and PBS at 4 °C to minimize nonspecific binding. Subsequently, the tissues underwent a 90-min incubation at room temperature with a biotin-conjugated secondary antibody diluted in PBS (Table 2). ExtrAvidin^®^-Peroxidase (Sigma-Aldrich, St. Louis, MO, USA), diluted to a ratio of 1:200 with PBS, was then administered and allowed to incubate at room temperature for 60 min.

For protein expression level determination, a chromogenic diaminobenzidine (DAB) substrate kit was employed (prepared immediately prior to use with 5 mL of distilled water, four drops of DAB, two drops of buffer, and two drops of hydrogen peroxide). The DAB substrate facilitated the development of a brown stain, enabling signal visualization. Negative control sections for each protein (NLRP3, ASC, caspases 1, 5, and 8, and interleukin IL-1β) underwent an identical procedure, with the primary antibody replaced by a blocking PBS solution. Hematoxylin staining with Carazzi hematoxylin for 15 min was employed to provide contrast in all histological samples.

### 4.4. Gene Expression Assessed by Real-Time Quantitative PCR

RNA extraction was conducted using the guanidinium thiocyanate–phenol–chloroform method, facilitating the assessment of mRNA expression levels for specific genes [57]. Complementary DNA (cDNA) was synthesized from RNA samples (concentration of 50 ng/µL) through reverse transcription (RT). During this procedure, each sample was mixed with an oligo-dT solution and subjected to denaturation at 65 °C for 10 min. Subsequently, a reverse transcription mix consisting of first-strand buffer, deoxyribonucleotides triphosphate, dithiothreitol, DNase- and RNase-free water, an RNase inhibitor, and a reverse transcriptase enzyme was added to each sample.

The reverse transcription process was carried out using a G-Storm GS1 thermal cycler. Samples were initially heated to 70 °C for 15 min to denature the reverse transcriptase enzyme, followed by gradual cooling to 4 °C. Subsequently, the samples underwent the synthesis of cDNA at 37 °C for 75 min. To verify the absence of genomic DNA contamination, a negative reverse transcription was performed by replacing the M-MLV RT enzyme with water devoid of DNases and RNases. The resulting cDNA was stored at −20 °C after being diluted 1:20 in water free from DNases and RNases.

Specific primers for selected genes (NLRP3, ASC, caspases 1, 5, and 8, and interleukin IL-1β) were designed using Primer-BLAST (v. 0.4.0) and AutoDimer (v 1.0.) online tools. The constitutively expressed TATA-box-binding protein (TBP) gene served as a normalization control [58]. Relative mRNA levels were quantified to measure gene expression units. RT-qPCR was conducted utilizing the relative standard curve method on a StepOnePlusTM System (Applied Biosystems; Thermo Fisher Scientific, Inc.). Each sample (diluted 1:20) was mixed with iQTM SYBR^®^ Green Supermix (Bio-Rad Laboratories, Inc., Hercules, CA, USA), forward and reverse primers, and RNase- and DNase-free water in a MicroAmp^®^ 96-well plate (Applied Biosystems; Thermo Fisher Scientific, Inc.). Thermocycling conditions involved multiple cycles of denaturation, annealing at different temperatures, and elongation. Additionally, a dissociation curve was generated for further analysis.

Fluorescence detection was conducted during the dissociation curve phases and at the end of each amplification cycle. To establish a standard curve encompassing information from the selected genes, a combination of samples was serially diluted as per the manufacturer’s instructions. TBP constitutive expression was included in each plate along with this curve. RT-qPCR was performed twice on each placental tissue sample, in accordance with previous research [10] (Table 3).

### 4.5. Histopathological Assessment

Examinations were conducted using a Zeiss Axiophot optical microscope (Zeiss GmbH, Jena, Germany). Each patient’s specimens from predetermined groups underwent assessment across five sections and ten randomly selected fields of view. Employing the immunoreactive score (IRS) framework established in previous studies, positive expression was defined as the presence of staining occupying at least 5% of the total examined sample [59]. Subsequently, two independent histologists assigned a score to each sample on a scale of 0 to 1 for minimal staining (25%), 2 to 4 for moderate staining (25–65%), and 3 to 4 for intense staining (65–100%).

### 4.6. Statistical Analysis

Data analysis was conducted utilizing the GraphPad Prism^®^ v6.0 software (GraphPad, Inc., San Diego, CA, USA). The normality of marker distributions was assessed through a Kolmogorov–Smirnoff test (all *p* < 0.001). Given the evident deviation from normality, non-parametric tests were employed, with data summarized using medians and interquartile ranges. The Mann–Whitney U test facilitated comparisons between the two groups. Significance thresholds were set at *p* < 0.05 (*), *p* < 0.01 (**), and *p* < 0.001 (***).

## 5. Conclusions

In the present investigation, a conspicuous upregulation in the genetic and protein manifestation of NLRP3 inflammasome constituents, including NLRP3, ASC, caspases 1, 5, and 8, alongside interleukin IL-1β, has been discerned. This observation implies a plausible pathophysiological implication of these elements within the placental tissue of individuals afflicted with CVD. Subsequent research endeavors should prioritize the assessment of potential translational interventions addressing this dysregulation in affected patient cohorts.

## Figures and Tables

**Table 1:** Clinical characteristics of participants enrolled in the study. The same cohort was also studied in previous research [56].

Clinical Features	CVD (n = 62)	HC (n = 52)
Median age (IQR), years	33 (22–40)	34 (27–41)
Median gestational age (IQR), weeks	40.5 (39–41.5)	41 (39–42)
Caesarean delivery, n (%)	12 (19.4)	9 (17.3)
Vaginal delivery, n (%)	50 (80.6)	43 (82.7)
CVD CEAP 1, n (%)	37 (59.7)	0 (0)
CVD CEAP 2, n (%)	21 (33.8)	0 (0)
CVD CEAP 3, n (%)	4 (6.5)	0 (0)
Prior pregnancies, n (%)	33 (53.2)	19 (36.5)
Prior abortions, n (%)	14 (22.6)	9 (17.3)
Regular periods of menstruation, n (%)	50 (80.6)	42 (80.7)
Sedentary profession, n (%)	41 (66.1)	40 (76.9)

**Table 2:** Antibodies for immunohistochemistry assays.

Antigen	Species	Dilution	Provider	Protocol Specifications
NLRP3	Rabbit Monoclonal	1:500	Abcam (Cambridge, UK) (ab263,899)	10 mM Sodium citrate pH = 6, before incubation with blocking solution
ASC	Rabbit monoclonal	1:250	Abcam (ab283,684)	100% Triton 0.1% in PBS for 10 min, before incubation with blocking solution
Caspase 1	Rabbit Polyclonal	1:500	Abcam (ab62,698)	EDTA pH = 9, before incubation with blocking solution
Caspase 5	Rabbit monoclonal	1:100	Abcam (ab40,887)	10 mM Sodium citrate pH = 6, before incubation with blocking solution
Caspase 8	Rabbit polyclonal	1:250	Abcam (ab25,901)	100% Triton 0.1% in PBS for 10 min, before incubation with blocking solution
IL-1β	Rabbit recombinant multiclonal	1:50	Abcam (ab283,818)	Not Specifications
IgG	Hybridoma Rabbit-Mouse monoclonal	1:1000	Sigma-Aldrich (RG96/B5283)	Not specifications

**Table 3:** Primers for RT-qPCR techniques.

Gene	Sequence Fwd (5′→3′)	Sequence Rev (5′→3′)	Temperature
TBP	TGCACAGGAGCCAAGAGTGAA	CACATCACAGCTCCCCACCA	60 °C
NLRP3	GCTGGCATCTGGATGAGGAA	GTGTGTCCTGAGCCATGGAA	61 °C
ASC	ATCCAGGCCCCTCCTCAG	AGAGCTTCCGCATCTTGCTT	60 °C
Caspase 1	GAAAAGCCATGGCCGACAAG	GCTGTCAGAGGTCTTGTGCT	57 °C
Caspase 5	TGTTAGCTATGGCTGAAGACAGT	TTGATGAGCCACGCGATTCT	58 °C
Caspase 8	GTCTGTACCTTTCTGGCGGA	CTCAGGCTCTGGCAAAGTGA	60 °C
IL-1β	AGCCATGGCAGAAGTACCTG	TGAAGCCCTTGCTGTAGTGG	60 °C
IL-18	GCTGAAGATGATGAAAACCTGGA	GAGGCCGATTTCCTTGGTCA	59 °C

## Data Availability

The data used to support the findings of the present study are available from the corresponding authors upon request.
